# Supplementation of L-Arginine, L-Glutamine, Vitamin C, Vitamin E, Folic Acid, and Green Tea Extract Enhances Serum Nitric Oxide Content and Antifatigue Activity in Mice

**DOI:** 10.1155/2020/8312647

**Published:** 2020-04-11

**Authors:** Yi-Ming Chen, Huashuai Li, Yen-Shuo Chiu, Chi-Chang Huang, Wen-Chyuan Chen

**Affiliations:** ^1^Health Technology College, Jilin Sport University, Changchun 130022, Jilin, China; ^2^Graduate Institute of Sports Science, National Taiwan Sport University, Taoyuan City 33301, Taiwan; ^3^Department of Orthopedics, Shuang Ho Hospital, Taipei Medical University, Taipei 23561, Taiwan; ^4^School of Nutrition and Health Sciences, College of Nutrition, Taipei Medical University, Taipei City 11031, Taiwan; ^5^Center for General Education, Chang Gung University of Science and Technology, Taoyuan 33301, Taiwan; ^6^Department of Otorhinolaryngology–Head and Neck Surgery, Sleep Center, Linkou-Chang Gung Memorial Hospital, Taoyuan 33301, Taiwan

## Abstract

It has been reported that abundant nitric oxide content in endothelial cells can increase exercise performance. The purpose of this study was to evaluate the potential beneficial effects of a combined extract comprising L-arginine, L-glutamine, vitamin C, vitamin E, folic acid, and green tea extract (LVFG) on nitric oxide content to decrease exercise fatigue. Male ICR (Institute of Cancer Research) mice were randomly divided into 4 groups and orally administered LVFG for 4 weeks. The 4-week LVFG supplementation significantly increased serum nitric oxide content in the LVFG-1X and LVFG-2X groups. Antifatigue activity and exercise performance were evaluated using forelimb grip strength, exhaustive swimming test, and levels of serum lactate, ammonia, glucose, and creatine kinase (CK) after an acute swimming exercise. LVFG supplementation dose-dependently improved exercise performance and nitric oxide content, and it dose-dependently decreased serum ammonia and CK activity after exhaustive swimming test. LVFG's antifatigue properties appear to manifest by preserving energy storage (as blood glucose) and increasing nitric oxide content. Taken together, our results show that LVFG could have the potential for alleviating physical fatigue due to its pharmacological effect of increasing serum nitric oxide content.

## 1. Introduction

Nitric oxide (NO) is known as the “endothelium-derived relaxing factor” for the maintenance of cardiovascular homeostasis. Nitric oxide synthases (NOS) are enzymes containing heme prosthetic groups, which are responsible for the synthesis of NO from L-arginine [1, 2]. Over the years, it has become increasingly evident that the decreased bioavailability of NO plays a role in several cardiovascular disorders such as atherosclerosis [3] and hypertension [4]. It is well known that exercise causes an increase in reactive oxygen production (ROS), particularly in active skeletal muscle. NO has been proposed to protect against cellular damage, often with the concomitant formation of superoxide/hydrogen peroxide. Hence, a synergistic relationship between the cytotoxic effects of nitric oxide and these active oxygen species is frequently assumed [5]. A previous study has demonstrated that the effect of aerobic exercise on endothelial function is mainly related to improved NO bioavailability due to increased production and/or decreased inactivation by superoxide [6]. Some studies suggest that L-arginine supplementation can reduce skeletal muscle damage after ischemia-reperfusion [7] and reduce oxidative stress and inflammation after exhaustive exercise in both young [8] and old rats [9]. To the best of our knowledge, research to date has shown that enhancement of exercise performance by using L-arginine alone as an ergogenic nutritional supplement is difficult [10]. This is in agreement with a previous hypothesis that it is the synergistic effect of various ingredients in sport nutritional supplements that may be responsible for reported improvements in exercise performance [11]. As a constituent of proteins, L-glutamine is the most abundant free amino acid in human muscle and plasma and is also an important vehicle for nitrogen transport [12]. Previous studies have claimed that glutamine supplements can benefit athletes by enhancing buffering capacity and improving high-intensity exercise performance [13].

Both L-arginine and L-glutamine are nonessential amino acids. Although they are not required for stimulating muscle protein synthesis [14], it does not mean that they are not important for maximizing training adaptation in athletes. Consumption of pre-workout liquid sports beverages has gained popularity over the past several years. Research has demonstrated that energy drinks are among the most popular dietary supplements consumed by young people in the United States [15]. In commercially available energy drinks, vitamin C, vitamin E, and green tea extract are among the more common ingredients. The primary purposes of ingesting energy drinks include enhancing workouts, improving sports performance, and facilitating faster training adaptation [16–18]. On the other hand, the ingestion of folic acid has been shown to improve blood flow via enhanced vascular conductance in the skeletal muscle of exercising aged humans [19].

Many researchers are interested in using the synergistic effects of various ingredients to delay fatigue and accelerate the elimination of fatigue-related metabolites [20]. To date, relatively few studies have directly addressed the antifatigue activity of L-arginine, L-glutamine, vitamin C, vitamin E, folic acid, and green tea extract complex (LVFG). In the current study, we used our established *in vivo* platform [21, 22] to evaluate the effects of LVFG supplementation on antifatigue activities and serum nitric oxide levels.

## 2. Methods

### 2.1. Preparation of LVFG Complex

A commercially available dietary supplement consisting of LVFG (L-arginine, L-glutamine, vitamin C, vitamin E, green tea extract, and folic acid complex) was provided by Pemey Bio-medical Co., Ltd. (Taichung, Taiwan). The LVFG contained 4 kcal/g with % (wt/wt) constituents as follows: 100% protein, 0% total fat, 0% saturated fat, 0% trans fat, 0% carbohydrate, and 0.0002% sodium. The amounts of L-arginine, L-glutamine, vitamin C, vitamin E, green tea extract, and folic acid in the LVFG were 350 mg/g, 100 mg/g, 25 mg/g, 5 mg/g, 15 mg/g, and 5 mg/g, respectively. The supplement was stored at room temperature and kept in a dark and dry cabinet. It was freshly prepared before each daily administration.

### 2.2. Animals and Experiment Design

Male ICR mice (8 weeks old) grown under specific pathogen-free conditions were purchased from BioLASCO (Yi-Lan, Taiwan). All mice were provided with a standard laboratory diet (No. 5001; PMI Nutrition International, Brentwood, MO, USA) and distilled water *ad libitum* and were housed on a 12-hour light/12-hour dark cycle at room temperature (22°C ± 1°C) and 50%–60% humidity. The Institutional Animal Care and Use Committee (IACUC) of the National Taiwan Sport University (NTSU) inspected all animal experiments, and this study conformed to the guidelines of protocol IACUC-10514 approved by the IACUC ethics committee. The 1X dose of LVFG used for humans is typically 3000 mg per day. The 1X mouse dose (615 mg/kg) we used was converted from a human-equivalent dose (HED) based on body surface area according to the US Food and Drug Administration formula: assuming a human weight of 60 kg, the HED for 3000 (mg)/60 (kg) = 50 × 12.3 = 615 mg/kg; the conversion coefficient 12.3 was used to account for differences in body surface area between mice and humans as previously described [23]. In total, 32 mice were randomly assigned to 4 groups (8 mice/group) for daily vehicle/LVFG oral gavage for 4 weeks. The 4 groups were the vehicle, 615 mg/kg (LVFG-1X), 1230 mg/kg (LVFG-2X), and 3075 mg/kg (LVFG-5X) groups. The vehicle group received the equivalent volume of solution based on individual body weight (BW). Mice were randomly housed in groups of 4 per cage.

### 2.3. Detection of Serum Nitric Oxide Content

The Total Nitric Oxide Assay Kit (Thermo Fisher, Catalog Number: EMSNOTOT, Austria) was used for the detection of serum nitric oxide content. The kit uses the enzyme nitrate reductase to convert nitrate (NO^3−^) to nitrite (NO^2−^). Nitrite is detected as a colored azo dye product of the Griess reaction that absorbs visible light at 540 nm. The total nitric oxide contributed by nitrate and nitrite in a system is measured as nitrite after converting all nitrate to nitrite [24].

### 2.4. Forelimb Grip Strength and Exhaustive Swimming

A low-force testing system (Model-RX-5, Aikoh Engineering, Nagoya, Japan) has been described in our previous study [25]. The swim-to-exhaustion test involves loads corresponding to 5% of the mouse BW attached to the tails to evaluate endurance times as previously described [20, 26].

### 2.5. Fatigue-Associated Biochemical Indices

The effects of LVFG on serum lactate, ammonia, glucose levels, and CK activity were evaluated after exercise. One hour after the last administration, a 15-minute swimming test was performed without weight-loading. Lactate, ammonia, glucose levels, and CK activity in the serum were determined with an autoanalyzer (Hitachi 7060, Hitachi, Tokyo, Japan). The other biochemical variables, as shown in Table 1, were measured with an autoanalyzer (Hitachi 7080) after 40 weeks of LVFG supplementation without exercise.

### 2.6. Tissue Glycogen Determination and Visceral Organ Weight

The stored form of glucose is glycogen, which exists mostly in liver and muscle tissues. Liver and muscle tissues were excised after the mice were euthanized and weighed for glycogen content analysis as described previously [21].

### 2.7. Histological Staining of Tissues

Different tissues were collected and fixed in 10% formalin after the mice were sacrificed. Following formalin fixation, the tissues were embedded in paraffin and cut into 4 *μ*m thick slices for histological and pathological evaluations. Tissue sections were then stained with hematoxylin and eosin and examined under a light microscope with a CCD camera (BX-51, Olympus, Tokyo, Japan) by a clinical pathologist.

### 2.8. Statistical Analysis

All data are expressed as mean ± SEM. Statistical differences among groups were analyzed by a one-way analysis of variance (ANOVA), and the Cochran-Armitage test was used for the dose-effect trend analysis. All statistics were calculated in SPSS version 18.0 (SPSS, Chicago, IL, USA), and *p* values < 0.05 were considered statistically significant.

## 3. Results

### 3.1. Morphological Data

The morphological data from each experimental group are summarized in Table 2. There were no significant differences in initial or final BW or in daily intake of diet and water among the vehicle, LVFG-1X, LVFG-2X, and LVFG-5X groups. We observed that LVFG supplementation had no effect on water and diet intake, with BW in each group steadily increasing throughout the experimental period (data not shown). We also observed no significant differences in the liver, kidney, heart, lung, epididymal fat pad (EFP), and muscle weights among the groups. However, we found the weight of the brown adipose tissue (BAT) to be significantly higher in the LVFG-2X and LVFG-5X groups (Δ1.13-fold, *p*=0.0103, and Δ1.15-fold, *p*=0.0063, respectively) than in the vehicle group. We also measured the effect of LVFG on the relative tissue weight. The relative BAT weights were higher in the LVFG-2X and LVFG-5X groups (Δ1.11-fold, *p*=0.0191, *p*=0.0311, respectively) than those in the vehicle group.

### 3.2. Effect of LVFG Supplementation on Exercise Performance and Serum Nitric Oxide (NO) Levels

**Figure 1:**
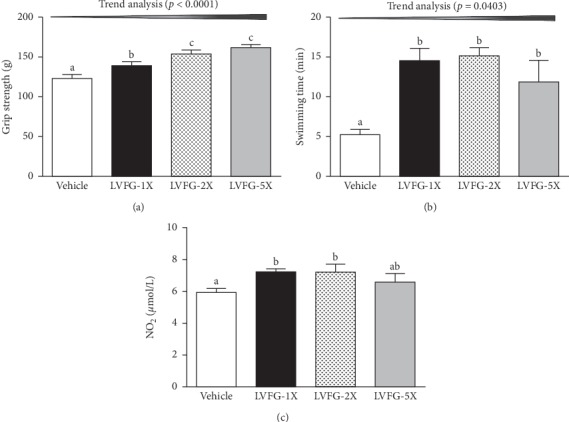
Effect of LVFG supplementation exercise performance. (a) Forelimb grip strength. (b) Swimming exercise performance: mice were subjected to an exhaustive swimming exercise with a load equivalent to 5% of the mouse's body weight attached to its tail, and exercise performance test male ICR mice were pretreated with vehicle or 615, 1230, and 3075 mg/kg LVFG (1X, 2X, and 5X, respectively) before undergoing a grip strength test and swimming test 1 h after the final administered dose. (c) Effect of LVFG on serum nitric oxide (NO) at rest: all mice were sacrificed and examined for nitric oxide levels after the final treatment. Data is expressed as mean ± SEM for *n* = 8 mice in each group. One-way ANOVA was used for the analysis, and different letters (a, b) indicate significant difference at *p* < 0.05.

As shown in Figure 1(a), the forelimb grip strength was higher in the LVFG-1X, LVFG-2X, and LVFG-5X groups than that in the vehicle group. Trend analysis showed that the grip strength dose-dependently increased with LVFG (*p* < 0.0001). Typically, a regulated training program is needed to obtain elevation in grip strength; however, our results indicated that LVFG treatment was able to improve grip strength even without training intervention. The swimming time was higher in all LVFG groups than that in the vehicle group (*p* < 0.001) (Figure 1(b)). Thus, the swimming time in the LVFG-1X, LVFG-2X, and LVFG-5X groups significantly increased (Δ2.78-fold, Δ2.89-fold, and Δ2.25-fold, respectively) compared with that in the vehicle group. In addition, a significant dose-dependent effect on swimming time was observed (*p*=0.0403). As shown in Figure 1(c), the serum nitric oxide levels in the vehicle, LVFG-1X, LVFG-2X, and LVFG-5X groups were 5.93 ± 0.24, 7.23 ± 0.20, 7.21 ± 0.49, and 6.60 ± 0.51 *μ*mol/L, respectively. Serum nitric oxide levels were significantly higher in the LVFG-1X and LVFG-2X groups (*p*=0.0225, *p*=0.0240, respectively) than those in the vehicle group.

### 3.3. Biochemistry Levels after Acute Exercise Challenge

**Figure 2:**
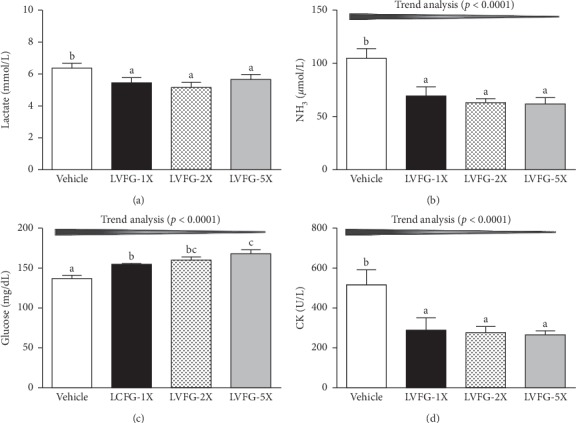
Effects of LVFG supplementation on serum (a) lactate, (b) ammonia, (c) glucose, and (d) creatine kinase (CK) levels after an acute exercise challenge. Mice were pretreated with the vehicle, 615, 1230, and 3075 mg/kg of LVFG for 4 weeks. One hour after the last treatment dose was administered, a 15-minute swimming test was performed without weight loading. Data is expressed as mean ± SEM of eight mice in each group. Columns with different letters (a, b) significantly differ by one-way ANOVA (*p* < 0.05).

Lactate accumulation and metabolic acidosis are cellular manifestations of fatigue. In the present study, the lactate levels in the vehicle, LVFG-1X, LVFG-2X, and LVFG-5X groups were 6.5 ± 0.3, 5.5 ± 0.3, 5.2 ± 0.2, and 5.7 ± 0.3 mmol/L, respectively. This corresponds to decreases in the LVFG-1X, LVFG-2X, and LVFG-5X groups (▽-14.86%, *p*=0.0110; ▽-19.66%, *p*=0.0011; and ▽-12.07%, *p*=0.0359, respectively) compared with the vehicle group (Figure 2(a)). This suggests that LVFG supplementation has the potential to increase the clearance or utilization of blood lactate during exercise.

The nitrogenous waste products of amino acid degradation are eliminated through the formation of urea and small amounts of ammonia [27]. Ammonia levels were significantly lower in the LVFG-1X, LVFG-2X, and LVFG-5X groups (▽-33.40%, *p*=0.0012; ▽- 39.71%, *p*=0.0002; and ▽-41.15%, *p*=0.0001, respectively) than those in the vehicle group (Figure 2(b)). In the trend analysis, serum ammonia levels decreased in a dose-dependent manner with increased LVFG dose (*p* < 0.0001), suggesting that continuous supplementation with LVFG for 4 weeks could mitigate ammonia accumulation during exercise.

Blood glucose level is an important index of performance maintenance during exercise. As exercise continues, there is an increase in glucose uptake and a decrease in intramuscular glucose concentration as the hexokinase inhibition is relieved by a lower glucose 6-phosphate (G-6-P) concentration [28]. Levels of serum glucose were higher in the LVFG-1X, LVFG-2X, and LVFG-5X groups (Δ1.14-fold, *p*=0.0001; Δ1.17-fold, *p* < 0.0001; and Δ1.23-fold, *p* < 0.0001, respectively) than those in the vehicle control (Figure 2(c)). Trend analysis showed dose-dependent increases in serum glucose level with increased LVFG supplementation (*p* < 0.0001).

Unusually high exercise volume can result in increased levels of creatine kinase (CK), indicating muscle damage and muscle fatigue [29]. Serum CK is an important clinical biomarker for muscle damage, such as muscular dystrophy, severe muscle breakdown, myocardial infarction, autoimmune myositis, and acute renal failure. CK activity was lower in the LVFG-1X, LVFG-2X, and LVFG-5X groups (▽-44.21%, *p*=0.0006; ▽-46.45%, *p*=0.0003; and ▽-48.50%, *p*=0.0002, respectively) than that in the vehicle group (Figure 2(d)). Our findings suggest that LVFG supplementation could ameliorate skeletal muscle injury induced by acute exercise challenge. Trend analysis showed that LVFG treatment had a significant dose-dependent effect on CK level (*p* < 0.0001). According to this data, provision of L-arginine and L-glutamine may minimize muscle damage.

### 3.4. Hepatic Glycogen Level

**Figure 3:**
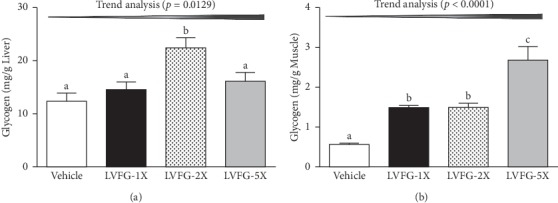
Effect of LVFG on (a) muscle and (b) liver glycogen levels at rest. Mice were pretreated with either vehicle, 615, 1230, or 3075 mg/kg of LVFG for 4 weeks. All mice were sacrificed and examined for glycogen levels in muscle and liver tissues 1 h after the final treatment. Data is expressed as mean ± SEM with *n* = 8 mice in each group. One-way ANOVA was used for the analysis, and different letters (a, b) indicate significant difference at *p* < 0.05.

The glycogen contents in the liver and muscle tissues of the mouse groups were examined (Figures 3(a) and 3(b)). The liver glycogen levels in the vehicle, LVFG-1X, LVFG-2X, and LVFG-5X groups were 12.41 ± 1.54, 14.63 ± 1.41, 22.46 ± 1.99, and 16.21 ± 1.61 mg/g liver, respectively. The LVFG-2X group showed a significantly higher (Δ1.81-fold, *p*=0.0001) liver glycogen level than that of the vehicle group. The muscle glycogen contents in the LVFG-1X, LVFG-2X, and LVFG -5X groups showed increases of 2.66-fold (*p*=0.0012), 2.66-fold (*p*=0.0012), and 4.79-fold (*p* < 0.0001) relative to that of the vehicle group. Trend analysis revealed that LVFG treatment had a significant dose-dependent effect on liver (*p*=0.0129) and muscle (*p* < 0.0001) glycogen levels. At the higher LVFG-5X doses, results also indicated that liver glycogen did not significantly increase, but the exercise performance was significantly elevated with LVFG supplementation. Some studies have shown an effect of glutamine supplementation in promoting glycogen synthesis in the first few hours of recovery after exercise [30].

### 3.5. Biochemical Markers

We observed that 4-week LVFG supplementation enhanced serum nitric oxide levels, increased exhaustive exercise challenge time, and improved antifatigue indicators, including lactate, ammonia, glucose, and CK levels. The liver and muscle glycogen storage capacities were both increased by LVFG. Further biochemical analyses carried out at the end of the study investigated whether the 4-week LVFG treatment affected other biochemical markers in the healthy mice. We examined tissue- and health status-related biochemical variables and major organs including the skeletal muscle, heart, kidney, and lung.

The results of the analysis are shown in Table 1. Levels of AST, ALT, creatinine, albumin, and glucose did not differ significantly among the groups. However, serum BUN levels were higher in the LVFG-2X and LVFG-5X groups than those in the vehicle group. Total protein (TP) levels were also significantly higher in the LVFG-1X, LVFG-2X, and LVFG-5X groups, respectively. Turning to the lipid profile, total cholesterol (TC) levels were significantly lower in the LVFG-1X (11.82%, *p*=0.0033) group, and serum triacylglycerol (TG) was lower by 21.68% (*p* < 0.0004) in the LVFG-5X group, compared with the vehicle group. The serum uric acid (UA) levels of the mice in the LVFG-1X, LVFG-2, and LVFG-5X groups were reduced by 40.43% (*p* < 0.0001), 44.68% (*p* < 0.0001), and 48.23% (*p* < 0.0001), respectively, as compared with the vehicle group.

**Figure 4:**
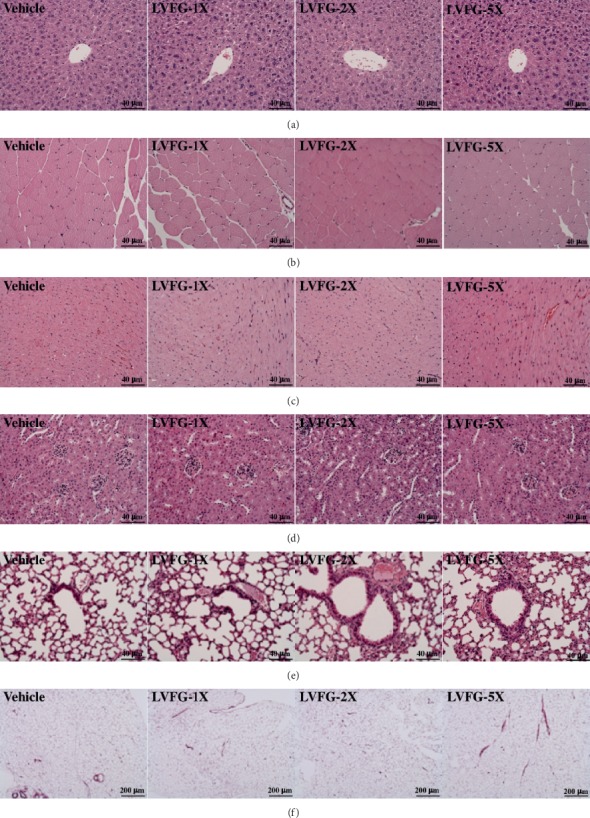
Effect of LVFG supplementation on the morphology of (a) liver, (b) skeletal muscle, (c) heart, (d) kidney, (e) epididymal fat pad, and (f) brown adipose tissue. Specimens were photographed with a light microscope (Olympus BX51). H&E stain, magnification: 400×. Scale bar, 10 *μ*m.

Additionally, our results also suggest that LVFG supplementation may have the potential to prevent lipid accumulation through the reduction of TC and TG. A previous study showed that a diet enriched with L-arginine lowered triglyceride [31] by decreasing TC and TG levels. We also found that total protein levels were significantly increased by LVFG treatment. The results of the histopathological examination of the major organs, including the liver, muscle, heart, kidney, and lung tissues, are shown in Figure 4. Histological observation of the sections showed that the liver, muscle, heart, kidney, lungs, EFP, and BAT of the mice supplemented with LVFG did not differ from those in the vehicle group.

## 4. Discussion

Nutrition plays an important role in exercise, training, and reduction of fatigue. Adequate hydration and electrolyte maintenance, appropriate energy intake, and adequate protein, carbohydrate, fat, vitamin, and mineral intake allow athletes to reap the maximal benefits from exercise. L-arginine and green tea extract activate BAT growth and development via mechanisms involving gene expression, nitric oxide signaling, and protein synthesis [32]. This activation has the potential to enhance the oxidation of energy substrates and reduce white fat accretion in the body. Arginine is required for the synthesis of protein and creatine, and its metabolism results in the production of nitric oxide. Very little scientific evidence has been reported to support the claims regarding arginine supplementation, such as the ability to elevate nitric oxide levels, increase muscle blood flow, and improve exercise performance. Some studies have found that supplementation of arginine alone has no effect on exercise performance [33]. Results of a present study suggest that supplementation of L-arginine and L-glutamine, in combination with vitamin C, vitamin E, folic acid, and green tea extract, is able to improve body composition and exercise performance. Our data suggest that different concentrations of LVFG may contribute differently to physiological activities, and the LVFG-2X (1230 mg/kg) dose may be the optimal range for explosiveness and endurance capacity. Interestingly, L-arginine or L-glutamine used alone had no significant effect on muscle performance, body composition, or muscle protein degradation in healthy adults [34]. Instead, our study suggests that continuous supplementation with LVFG for 4 weeks could increase serum glucose levels and improve glucose uptake capacity toward beneficial antifatigue activity.

Athletes may significantly reduce their muscle glycogen stores during exercise, leading to muscular fatigue [35]. Fully replacing the muscle glycogen stores before a subsequent bout of exercise or competition may prolong the time until fatigue and improve performance [36]. Our data suggests that different concentrations of LVFG may contribute differently to enhancing glycogen content and that a 1230 mg/kg dose may be the most appropriate for optimizing liver and muscle glycogen content. LVFG supplementation helped to increase muscle glycogen storage in the mice, leading to enhanced energy utilization.

During exercise, nitric oxide levels are naturally increased and more blood can flow through the arteries and arterioles for the purpose of delivering oxygen and fuel substrates to the working skeletal muscles. In previous research, it has been established that exercise induces iNOS expression and causes low concentrations of nitric oxide in humans [37]. Heavy physical exercise induces an immune response that in turn induces the expression of iNOS [38]. Therefore, nitric oxide concentration and enhanced iNOS expression are possible mechanisms of cell damage after exercise.

It has been hypothesized that, by elevating nitric oxide levels, arginine supplementation is beneficial for enhancing sport performance or maximizing training adaptations for athletes or physically active individuals. However, previous studies on arginine supplementation have failed to show any effect or beneficial outcomes [10]. Herein, our current study demonstrates that L-arginine, in combination with L-glutamine, vitamin C, vitamin E, folic acid, and green tea extract, increases serum nitric oxide and enhances sports performance. We found enhanced serum total protein (TP) content with LVFG supplementation, suggesting that the nonessential amino acids L-arginine and L-glutamine stimulated muscle protein synthesis [39]. However, this enhancement in TP content was not reflected as increased muscle growth in our study. Nevertheless, we still recommend that L-arginine and L-glutamine be added to the complex to maximize training adaptations in athletes.

## 5. Conclusions

In the current study, we found that 4-week LVFG supplementation significantly increased the BAT weight in the LVFG-treated groups and showed beneficial effects on the lipid profile. Exercise performance was significantly improved in the LVFG-2X group. In addition, exercise-induced fatigue-related parameters, including lactate, ammonia, glucose, and CK levels, were positively modulated by LVFG supplementation and dose-dependently for ammonia, glucose, and CK. With regard to serum nitric oxide levels, we also found that the LVFG-2X (1230 mg/kg) dose may be the optimal dose for increasing levels of nitric oxide. Taken together, the above findings suggest that LVFG-2x may be a potential ergogenic aid to increase nitric oxide levels, increase glycogen storage, and improve exercise performance. In conclusion, LVFG may have direct benefits for athletes by enhancing sports performance and/or maximizing training adaptations.

Values are expressed as mean ± SEM for *n* = 8 mice in each group. Values in the same line with different superscripts letters (a, b, c) differ significantly by one-way ANOVA (*p* < 0.05). Muscle mass includes both gastrocnemius and soleus muscles at the back part of the lower legs. EFP: epididymal fat pad; BAT: brown adipose tissue.

## Figures and Tables

**Table 1:** Biochemical analysis of mice subjected to LVFG supplementation at the end of the study.

Parameter	Vehicle	LVFG -2X	LVFG-5X	LVFG-1X	Trend analysis
AST (U/L)	85 ± 6	85 ± 8	90 ± 6	75 ± 3	0.3792
ALT (U/L)	53 ± 5	54 ± 5	46 ± 4	47 ± 3	0.3529
BUN (mg/dL)	24.4 ± 0.5	25.3 ± 1.3	27.4 ± 0.8	31.7 ± 0.9	<0.0001
Creatinine (mg/dL)	0.32 ± 0.02	0.35 ± 0.02	0.31 ± 0.01	0.32 ± 0.01	0.4556
UA (mg/dL)	1.41 ± 0.09	0.84 ± 0.07	0.78 ± 0.04	0.73 ± 0.03	<0.0001
TC (mg/dL)	162 ± 5^b^	143 ± 4^a^	162 ± 4^b^	174 ± 4^bc^	0.0287
TG (mg/dL)	179 ± 6^b^	168 ± 9^b^	162 ± 6^b^	140 ± 6^a^	<0.0001
TP (g/dL)	5.5 ± 0.2^a^	6.3 ± 0.1^b^	6.3 ± 0.1^b^	6.2 ± 0.1^b^	0.0372
Albumin (g/dL)	3.6 ± 0.1	3.6 ± 0.0	3.6 ± 0.0	3.6 ± 0.0	0.4637
Glucose (mg/dL)	148 ± 4	150 ± 4	153 ± 3	148 ± 7	0.9310

Values are mean ± SEM for *n* = 8 mice per group. Values in the same line with different superscripts letters (a, b, c) differ significantly (*p* < 0.05) by one-way ANOVA. AST, aspartate aminotransferase; ALT, alanine aminotransferase; BUN, blood urea nitrogen; UA, uric acid; TC, total cholesterol; TG, triacylglycerol; TP, total protein.

**Table 2:** General characteristics of mice with LVFG supplementation.

Characteristic	Vehicle	LVFG-1X	LVFG-2X	LVFG-5X	Trend analysis
Initial BW (g)	34.59 ± 0.77	35.04 ± 0.78	35.31 ± 0.40	34.64 ± 0.67	0.8701
Final BW (g)	36.91 ± 0.60	37.36 ± 0.74	37.38 ± 0.49	37.73 ± 0.51	0.2850
Food intake (g/day)	6.18 ± 0.07	6.12 ± 0.11	6.18 ± 0.04	6.16 ± 0.06	0.5236
Water intake (g//day)	6.77 ± 0.06	6.79 ± 0.10	6.82 ± 0.12	6.79 ± 0.09	0.2626
Liver (g)	2.05 ± 0.02	2.05 ± 0.06	2.09 ± 0.02	2.04 ± 0.05	0.7861
Kidney (g)	0.57 ± 0.01	0.59 ± 0.03	0.56 ± 0.02	0.57 ± 0.01	0.9738
EFP(g)	0.54 ± 0.07	0.50 ± 0.05	0.53 ± 0.02	0.53 ± 0.02	0.1256
Heart (g)	0.23 ± 0.01	0.23 ± 0.01	0.23 ± 0.01	0.23 ± 0.01	0.4543
Lung (g)	0.22 ± 0.00	0.21 ± 0.01	0.22 ± 0.00	0.22 ± 0.01	0.8911
Muscle (g)	0.39 ± 0.01	0.38 ± 0.01	0.39 ± 0.00	0.38 ± 0.01	0.8184
BAT (g)	0.11 ± 0.00^a^	0.11 ± 0.00^a^	0.13 ± 0.00^b^	0.13 ± 0.01^b^	<0.0001
Relative liver weight (%)	5.56 ± 0.10	5.51 ± 0.16	5.61 ± 0.10	5.40 ± 0.08	0.5681
Relative kidney weight (%)	1.54 ± 0.03	1.57 ± 0.07	1.50 ± 1.52	1.52 ± 0.03	0.5037
Relative EFP weight (%)	1.46 ± 0.17	1.33 ± 0.13	1.42 ± 0.06	1.41 ± 0.06	0.2622
Relative heart weight (%)	0.63 ± 0.02	0.61 ± 0.02	0.62 ± 0.02	0.59 ± 0.02	0.1839
Relative lung weight (%)	0.58 ± 0.01	0.57 ± 0.02	0.58 ± 0.01	0.58 ± 0.02	0.8147
Relative muscle weight (%)	1.05 ± 0.02	1.01 ± 0.04	1.05 ± 0.02	1.01 ± 0.03	0.5788
Relative BAT weight	0.30 ± 0.01^a^	0.30 ± 0.01^a^	0.34 ± 0.01^b^	0.34 ± 0.01^b^	0.0008

## Data Availability

The data used to support the findings of this study are included within the article.

## Abbreviations

LVFG: L-arginine, L-glutamine, vitamin C, vitamin E, folic acid, and green tea extract
NO: Nitric oxide
NOS: Nitric oxide synthases
ROS: Reactive oxygen production
NO^3−^: Nitrate
NO^2−^: Nitrite
EFP: Epididymal fat pad
BAT: Brown adipose tissue
AST: Aspartate aminotransferase
ALT: Alanine aminotransferase
BUN: Blood urea nitrogen
UA: Uric acid
TC: Total cholesterol
TG: Triacylglycerol
TP: Total protein
iNOS: Inducible nitric oxide synthase.
